# Are two better than one? VALIFT: video-assisted ligation of the intersphincteric fistula tract—a combination of two minimally invasive techniques for treatment of transsphincteric perianal fistulas

**DOI:** 10.1007/s10151-019-1925-3

**Published:** 2019-02-08

**Authors:** Michal Romaniszyn, Piotr Julian Walega

**Affiliations:** 1 Department of Medical Education, Jagiellonian University Medical College, Krakow, Poland; 2 3rd Department of General Surgery, Jagiellonian University Medical College, Krakow, Poland

## Introduction

Ligation of the intersphincteric fistula tract (LIFT) is one of many methods for treating transsphincteric anal fistulas. During this procedure, the fistula tract is dissected and ligated in the intersphincteric space, to close its lumen and prevent the fistula from recurring. The main advantage of this procedure over classic fistulectomy or fistulotomy operations is that both internal and external sphincters are left intact [1] and the risk of fecal incontinence after the operation is minimal. However, as in most techniques used to treat perianal fistulas, results vary depending on the complexity of the fistula. Due to this variability, many surgeons develop modifications of this technique, to achieve better results [2].

Video-assisted anal fistula treatment (VAAFT) was supposed to provide minimally invasive treatment by cauterization of the fistula tract from inside, under direct vision by means of a rigid small-caliber fistuloscope [3]. The early results in the literature were promising; however, later studies gave mixed results and we were not able to reproduce completely satisfactory results [4]. As most authors emphasize that the results of this endoscopic treatment depend on the method of internal opening closure during VAAFT (stapler, suture, advancement flap) [5], tight ligation of the fistula tract right after the internal opening (exactly as in the LIFT procedure), might give better results, than other methods of closure.

According to some researchers, recurrences after the LIFT procedure may be caused by omitted side branches, or by improper fistula tract identification in the intersphincteric space, thus leading to recurrence in form of a persistent transphincteric tract, downstaging to a intersphincteric fistula, or as a residual external tract [6]. A hypothesis arose, that the diagnostic value of fistuloscopy may improve the results of the LIFT procedure.

As our unit is experienced in both the LIFT technique, and the VAAFT procedure, we designed a pilot feasibility study in 2014 to combine both methods, LIFT and VAAFT, in an attempt achieve better results than in VAAFT alone, and to potentially increase the healing rates of the standard LIFT procedure, especially in patients with complex perianal fistulas.

## Materials and methods

We compared the results of video assisted LIFT (VALIFT) with standard LIFT in patients operated upon by the same team of surgeons, prior to introduction of the fistuloscope. Additionally, we compared the data with a historical database of patients operated upon in our department using VAAFT alone [4].

**Fig. 1:**
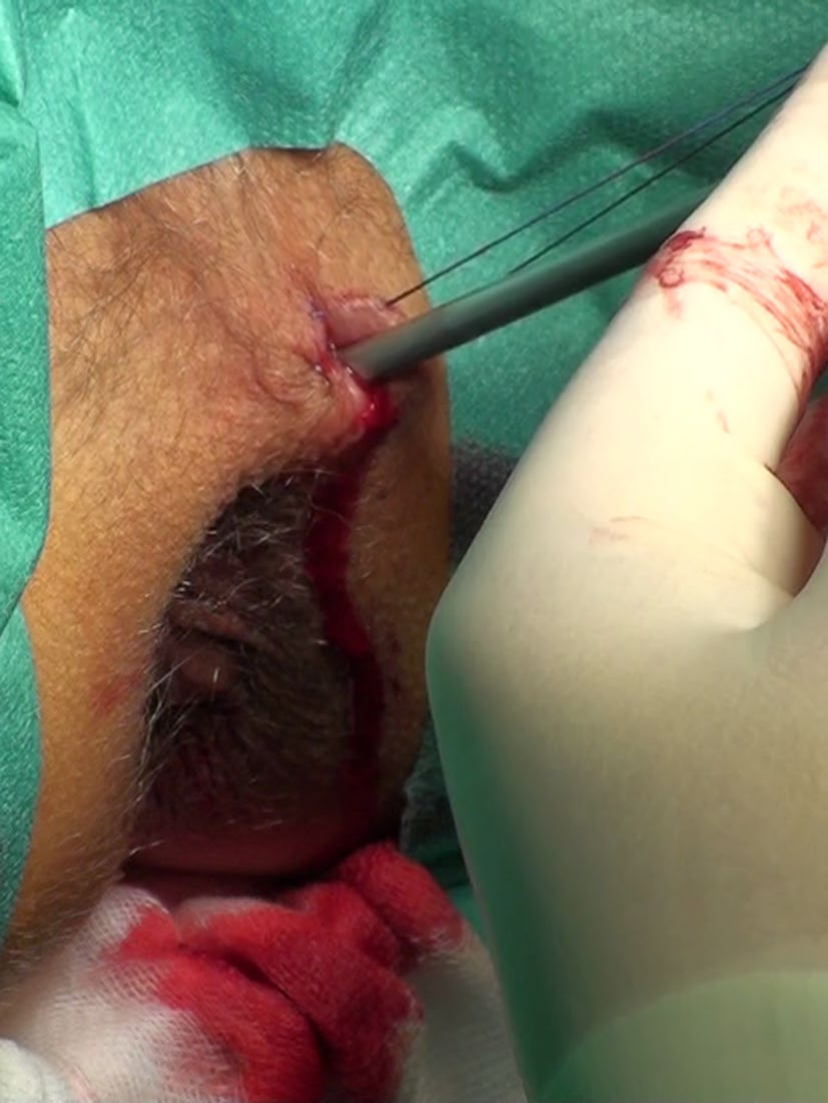
Identification the tract of the fistula with the fistuloscope

**Fig. 2:**
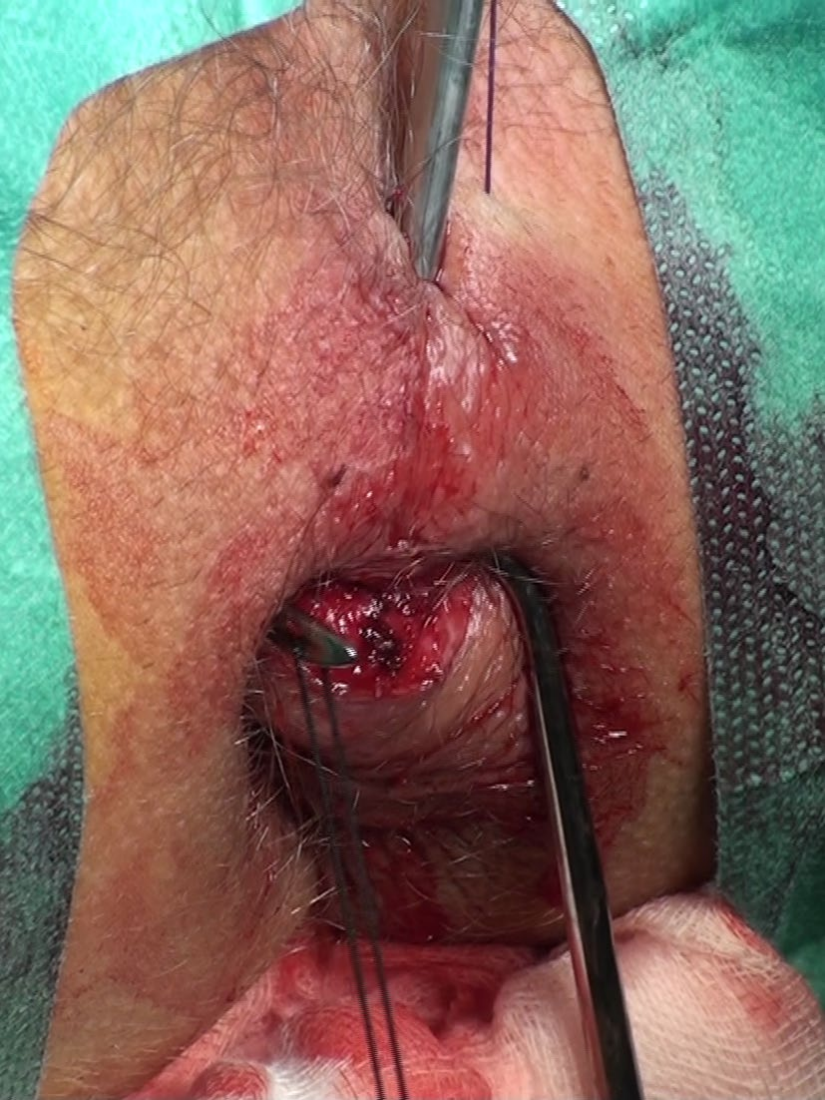
Dissected intersphincteric space, ligation of the intersphincteric part of the fistula tract

**Fig. 3:**
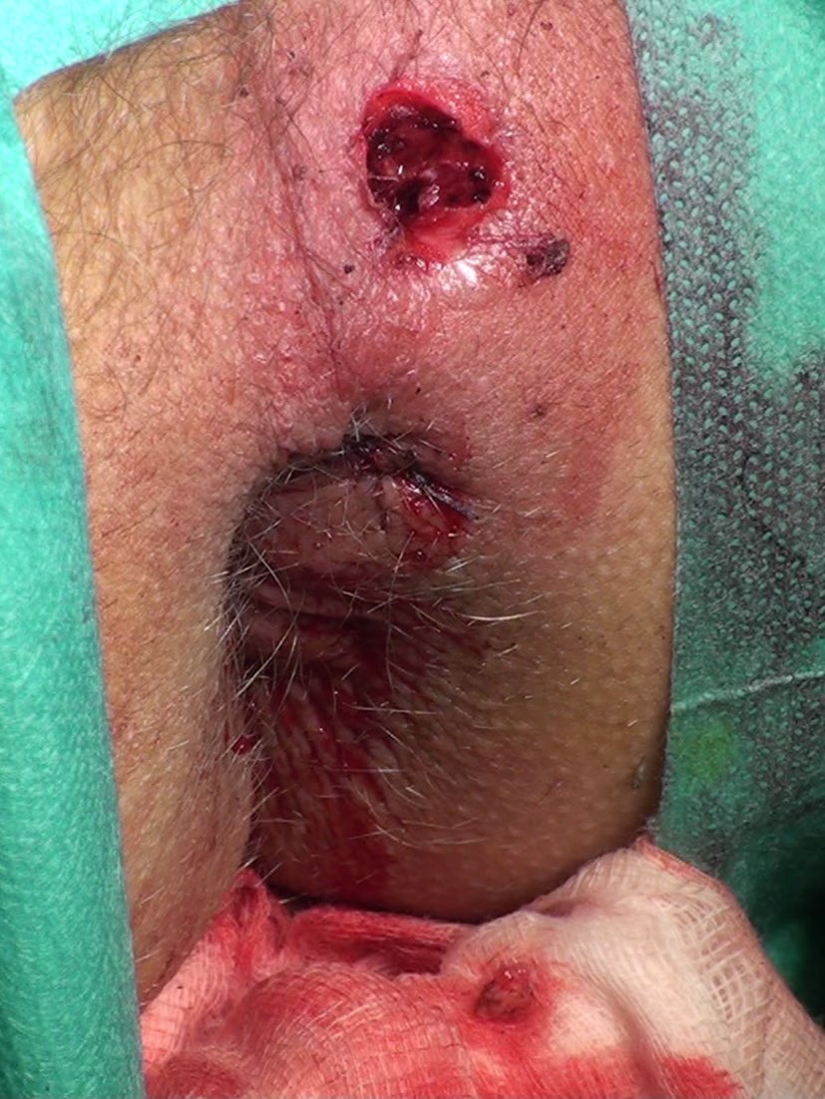
Surgical wound after the procedure

Twenty-four consecutive patients had the standard LIFT procedure (LIFT Group). The group consisted of 22 males and 2 females, with a mean age of 44.5 years (range 25–65 years). The 16 subsequent consecutive patients were operated on using the VALIFT technique. The VALIFT Group consisted of 12 male and 4 female patients with a mean age of 39.8 years (24–74 years). The demographical structure of both groups was comparable (*p* > 0.05). In the VALIFT Group, instead of using a standard fistula probe, a fistuloscope was inserted through the external opening to identify the tract of the fistula, additional branches (if any) and the internal opening (Fig. 1). Skin was then cut circumferentially in the intersphincteric groove, over the fistula tract. The intersphincteric space was dissected and the fistula tract was easily isolated, with the fistuloscope shaft supporting it from the inside. The external part of the tract (lateral from the external sphincter) and electrocautery was used on all its branches under direct vision, as in the VAAFT procedure. After putting ligatures around the tract, the fistuloscope was retracted and the fistula tract was ligated in the intersphincteric space and cut between ligations (Fig. 2). The internal opening was curreted and additionally closed using a “figure-of-eight” absorbable suture. The wound in the intersphincteric groove was sutured using interrupted absorbable sutures, the external opening was then cut out and the wound left open for drainage (Fig. 3). Patients attended regular follow-up visits, weekly for a month, then every 2 weeks. According to protocol, the patients with recurrences were excluded from further follow-up and qualified for secondary procedures. Patients with persistent fistulas who were qualified for secondary procedures were also excluded from further follow-up.

### Statistical analysis

The data were analyzed in StatSoft STATISTICA 13, using Pearson’s Chi^2^ test performed on a contingency cross-tabulation matrix. Additionally, the data were compared to our database on VAAFT [4], using the same statistical methods.

## Results

With the help of the fistuloscope, the operating surgeon was able to easily identify the fistula tract and the internal opening in all cases. In one patient in the VALIFT group an additional branch of the fistula tract was found which was not detected in preoperative evaluation by clinical examination, magnetic resonance imaging or endoanal ultrasound. The mean operating time for VALIFT was 71 min (range 45–90 min). In comparison, mean operating time for the standard LIFT procedure was 50 min (range 20–85 min). All patients were discharged from the hospital the day following the operation.

In the VALIFT group 14 patients (87.5%) achieved primary healing, and in the LIFT group 21 patients healed (also 87.5%, *p* > 0.05). In the VALIFT group mean time for healing was 59 days (range 29–116 days), whereas in the LIFT group mean time for healing was 71 days (range 18–155 days). The difference was not statistically significant (*p* > 0.05).

During follow-up, there were 2 cases of recurrence in the VALIFT group, occurring 37 and 42 days after initial healing, with the fistula penetrating through the wound in the intersphincteric groove in both cases. There was also a third recurrence 7 months after the procedure (in a form of a transphincteric fistula). In the LIFT group there were 5 recurrences and mean time to recurrence was 78 days (43–118 days). All recurrences in this group were in the form of a intersphincteric fistula (through the incision in the intersphincteric groove). The overall success rate during the follow-up was 68.75% in the VALIFT group and 66.67% in the LIFT group. The difference was not statistically significant (*p* > 0.05). There were no complications related to either LIFT or VALIFT procedure, nor did any of the patients report any problems with soiling or incontinence.

The statistical analysis of historical data on VAAFT (54.41% success rate) compared to VALIFT (68.75%) showed no statistical significance, despite the evident difference in raw numbers.

## Discussion

Despite high hopes, VALIFT did not increase healing rates over LIFT, with a significant increase of the cost of the procedure due to expensive equipment (about 7000 EUR in 2018). However, VALIFT seemed superior to VAAFT, based on our comparison with historical data (68.75% vs 54.41% in comparison of VALIFT vs VAAFT [4]) but this did not reach statistical significance. As the LIFT patients also had relatively higher healing rates than VAAFT patients, again not reaching statistical significance, probably due to small sample size comparison of LIFT and VAAFT needs further investigation.

The authors are aware that the major drawback of this study is the relatively small group of patients. Also, no there was no randomization regarding the choice of the procedure in each patient (LIFT, VAAFT or VALIFT). Therefore, there is a need for a randomized trial comparing LIFT, VAAFT, and VALIFT in larger groups of patients.

## Conclusions

Although the VALIFT technique seemed promising, it did not increase healing rates in our patients, compared to classical LIFT. Therefore, the additional cost of the fistuloscopy equipment used during the LIFT operation is not justified. There is a need for a randomized trial comparing LIFT, VAAFT, and VALIFT in larger groups of patients.
